# 

**Published:** 1894-04-28

**Authors:** 


CONTENTS.

The Place of Medicine in Civilisation - 67

Around the Hospitals - 68

Modern Medico-Psychology and Psychiatry. IV. - 69

Practical Aspects of Medical Science - 70

Artificial Respiration in Chloroform Collapse. By Sir Benjamin Ward Richardson, M.D., F.R.S. - 71

Annotations.—Addenbrooke's Hospital, Cambridge—A Village Doctor: New Style—Public Schools: Facts for Parents and the Press - 73

The Institutional Workshop—

Hospital Finance. VI.—Prospects for the Future - 83

Practical Departments - 84

Hospital Festivals, Meetings, &c. - 85

The Field of Science - 72

Notes and News - 74

Medical Progress and Hospital Clinics—

On Temperature Charts - 75

The Diagnostic Significance of Certain Signs and Symptoms in Joint Disease - 76

Medical Progress—Pulmonary Tuberculosis (continued) - 77

Progress in Surgery—Peritonitis - 79

Diseases of the Eye - 81

New Drugs and Praparations - 82

New Appliances and Things Medical - 82

The Book World of Medicine and Science - 87

Editor's Letter-Box—Scraps and Gleanings - 87

EXTRA SUPPLEMENT.—THE NURSING MIRROR.

News from the Nursing World.—"The Hospital" Convalescent Fund—Useful Information—A Brilliant Success—An English Heroine—On the River—District Nursing in Carlisle—Wise Miners—A Volunteer Wanted—The late Miss Mary Grenfell—The Lansdowne Hospital—Burglars and Nurses—The Royal British Nurses' Association - xxxi
On General Nursing. IX.—Pneumonia (continued) - xxxiii
The Friends and Foes of Nurses - xxxiv
The Ethics of Nursing. II. - xxxv
The Nursing of Army Sisters. II. - xxxvi
Hospital Treatment - xxxvi
Women's Work. How to Become a Cookery Teacher - xxxvii
The Hospital for Sick Children - xxxviii
The Great Northern Central Hospital - xxxviii
The Late Mrs. Wardroper—Appointments - xxxviii
Everybody's Opinion—For Reading to the Sick - xxxix
The Muses' Looking-Glass—Wanderings in Italy: - xl
Where to Go—Notes and Queries - xl

